# High Performance Implementation of 3D Convolutional Neural Networks on a GPU

**DOI:** 10.1155/2017/8348671

**Published:** 2017-11-08

**Authors:** Qiang Lan, Zelong Wang, Mei Wen, Chunyuan Zhang, Yijie Wang

**Affiliations:** ^1^College of Computer, National University of Defense Technology, Changsha 410073, China; ^2^National Key Laboratory of Parallel and Distributed Processing, Changsha 410073, China

## Abstract

Convolutional neural networks have proven to be highly successful in applications such as image classification, object tracking, and many other tasks based on 2D inputs. Recently, researchers have started to apply convolutional neural networks to video classification, which constitutes a 3D input and requires far larger amounts of memory and much more computation. FFT based methods can reduce the amount of computation, but this generally comes at the cost of an increased memory requirement. On the other hand, the Winograd Minimal Filtering Algorithm (WMFA) can reduce the number of operations required and thus can speed up the computation, without increasing the required memory. This strategy was shown to be successful for 2D neural networks. We implement the algorithm for 3D convolutional neural networks and apply it to a popular 3D convolutional neural network which is used to classify videos and compare it to cuDNN. For our highly optimized implementation of the algorithm, we observe a twofold speedup for most of the 3D convolution layers of our test network compared to the cuDNN version.

## 1. Introduction

Convolutional neural networks have proven advantages over traditional machine learning methods on applications such as image classification [1–4], tracking [5, 6], detection [7–11]. However, the primary downside of convolutional neural networks is the increased computational cost. This becomes especially challenging for 3D convolution where handling even the smallest instances requires substantial resources.

3D convolutional neural networks have recently come to the attention of the scientific community. In [12], a database for 3D object recognition named ObjectNet3D is presented. The database focuses on the problem of recognizing the 3D pose and the shape of objects from 2D images. Another repository of 3D CAD models of objects is ShapeNet [13]. In [14], the authors propose VoxNet, a 3D convolutional neural network, to solve the robust object recognition task with the help of 3D information, while the authors of [15] propose a 3D convolutional neural networks for human-action recognition.

In the light of these successful applications, it is worthwhile to explore new ways of speeding up the 3D convolution operation. In this paper we do so by deriving the 3D convolution forms of the minimal filtering algorithms invented by Toom and Cook [16] and generalized by Winograd [17]. Our experiments show this algorithm to be very efficient in accelerating 3D convolutional neural network in video classification applications.

## 2. Related Work

Many approaches aim to directly reduce the computational cost within CNN. In [18], the authors analyse the algebraic properties of CNNs and propose an algorithmic improvement to reduce the computational workload. They achieve a 47% reduction in computation without affecting the accuracy. In [19], convolution operations are replaced with pointwise products in the Fourier domain, which can reduce the amount of computation significantly. Reference [20] evaluates two fast Fourier transform (FFT) convolution implementations, one based on Nvidia cuFFT [21] and the other based on Facebook's FFT implementation. The FFT method can achieve an obvious speeding up of performance when the filter size is large, and the disadvantage of the FFT method is that it consumes much more memory than the standard method.

In [22], the authors use WMFA (Winograd Minimal Filter Algorithm) [17] to implement the convolution operation. In theory, fewer multiplications are needed in the WMFA, while not much extra memory is needed. WMFA is easy to parallelize; Lavin and Gray [22] implemented the algorithm on GPU, and they achieved better performance than the fastest cuDNN library. In [23], the authors show a novel architecture implemented in OpenCL on an FPGA platform; the algorithm they use to do the convolution is WMFA, which significantly boosts the performance of the FPGA. However, both works implemented 2D convolutional neural networks.

In this paper, we make four main contributions. Firstly, we derive the 3D forms of WMFA and design detailed algorithm to implement 3D convolution operation based on 3D WMFA. Secondly, we analyse the arithmetic complexity of 3D WMFA and prove 3D WMFA method can reduce computation in theory. Thirdly, we implement 3D WMFA for GPU platform and propose several optimization techniques to improve the performance of 3D WMFA. Finally, we evaluate the performance of 3D convolutional neural networks based on several implementations and prove the advantage of our proposed 3D WMFA method.

## 3. Fast 3D Convolution Algorithm

### 3.1. Preliminary: 3D Convolutional Neural Networks

For the 2D convolution, kernels have fixed width and height, and they are slid along the width and height of the input feature maps. For the 3D convolution, both feature maps and kernels have depth dimension, and the convolution also needs to slide along the depth direction. We can compute the output of a 3D convolutional layer using the following formula:

(1) $$\begin{array}{c} Y_{i,k,x,y,z}=\overset{C-1}{\underset{c=0}{\sum }}\;\overset{T-1}{\underset{t=0}{\sum }}\;\overset{R-1}{\underset{r=0}{\sum }}\;\overset{S-1}{\underset{s=0}{\sum }}I_{i,c,x+t,y+r,z+s}F_{k,t,r,s,c}, \end{array}$$

where *Y*_*i*,*k*,*x*,*y*,*z*_ represents the result of a convolution operation at the *k*th channel feature and *I*_*i*,*c*,*x*+*t*,*y*+*r*,*z*+*s*_ is one of the input features, while *F*_*k*,*t*,*r*,*s*,*c*_ is one of the filters. Equation (1) represents a direct convolution method, which requires intensive computability. The detailed arithmetic complexity of this method is shown in Section 3.3.

### 3.2. 3D WMFA

We introduce a new, fast algorithm to compute a 3D convolutional layer. The algorithm is based on WMFA. In order to introduce the 3D WMFA, firstly, we will give a simple introduction to the 1D WMFA. WMFA computes output with a tile size of *m* each time; we use *F*(*m*, *r*) to represent the output tile and *r* is the filter size. According to the definition of convolution, 2 × 3 = 6 multiplications are required to compute *F*(2,3), but we can reduce the number of multiplications to do the convolution if we use the following WMFA:

(2) $$\begin{array}{c} F\left(\;2,3\;\right)=\left[\begin{array}{ccc} i_{0} & i_{1} & i_{2}\\ i_{1} & i_{2} & i_{3} \end{array}\right]\left[\begin{array}{c} f_{0}\\ f_{1}\\ f_{2} \end{array}\right]=\left[\begin{array}{c} m_{1}+m_{2}+m_{3}\\ m_{2}-m_{3}-m_{4} \end{array}\right], \end{array}$$

where

(3) m1=i0−i2f0,m2=i1+i2f0+f1+f22,m4=i1−i3f2,m3=i2−i1f0−f1+f22.

The number of multiplications needed is *μ*(*F*(2,3)) = 2 + 3 − 1 = 4; however, four additions are needed to transform the input image, three additions to transform the filter, and four additions to transform the result of the dot product. We can use a matrix form to represent the computation:

(4) $$\begin{array}{c} Y=A^{T}\left[\begin{array}{c} \left(B^{T}i\right)⊙\left(Gf\right) \end{array}\right]. \end{array}$$

We call the *A*^*T*^, *G*, and *B*^*T*^ transform matrices, and the values of the transforming matrices are

(5) BT=10−1001100−110010−1,G=10012121212−1212001,AT=111001−1−1.

In (4), $i={\left[\begin{array}{cccc} i_{0} & i_{1} & i_{2} & i_{3} \end{array}\right]}^{T}$ and $f={\left[\begin{array}{ccc} f_{0} & f_{1} & f_{2} \end{array}\right]}^{T}$ represent the input tile and filter tile, respectively. As described in [22], the format of the 2D WMFA is as follows:

(6) $$\begin{array}{c} Y=A^{T}\left[\begin{array}{c} \left[\begin{array}{c} GfG^{T} \end{array}\right]⊙\left[\begin{array}{c} B^{T}iB \end{array}\right] \end{array}\right]A, \end{array}$$

where *f* is the filter with size *r* × *r* and *i* is the image with size (*m* + *r* − 1)×(*m* + *r* − 1). To compute *F*(2 × 2,3 × 3), we need 4 × 4 = 16 multiplications; however, 4 × 9 = 36 multiplications are needed according to the convolution definition. Therefore, 2D WMFA can reduce the number of multiplications by a factor of 36/16 = 2.25 at the cost of increasing 32 additions in the data transformation stage, 28 floating point instructions at the filter transformation stage, and 24 additions at the inverse transformation stage. For a convolutional layer, the number of input channels and number of output channels are large, which means the input channels need to convolve different filters, so the transformed input tile can be reused as many times as the number of output channels. Each filter needs to be slid in *x* and *y* direction of input channel during convolution, so each transformed filter is reused as many times as the number of subtiles of input channel. And since the output tile is reduced along the input channels, the inverse transformation is done after reduction; then the number of inverse transformation is determined by the number of output channels. Therefore, the cost of data transformation stage, filter transformation stage, and the inverse transformation stage keep low in real convolutional layer implementation.

We can also apply the 3D WMFA to 3D convolution. To compute *F*(2 × 2 × 2,3 × 3 × 3), we apply the 3D Winograd transformation to the input tile and filter tile and apply 3D Winograd inverse transformation to the dot product of the transformed input image tile and the transformed filter tile.  Algorithm 1 is a general form of the 3D Winograd transformation. In the algorithm, *T*_*m*_ is the transformation matrix; the transformation matrix can be *G* applied to transform the filter tile or *B*^*T*^ applied to transform the input image tile. The dot product of the transformed input image tile and transformed filter tile will be accumulated along the *C* channels, which can be converted to a matrix multiplication similar to the description in [22]

(7) Yi,x,y,z,k=∑c=0C−1Ii,c,x,y,z∗Fk,c=∑c=0C−1ATUk,c⊙Vc,i,x,y,zA=AT∑c=0C−1Uk,c⊙Vc,i,x,y,zA.

Consider the sum

(8) $$\begin{array}{c} M_{k,i,x,y,z}=\overset{C-1}{\underset{c=0}{\sum }}U_{k,c}⊙V_{c,i,x,y,z}. \end{array}$$

The previous equation can be divided into several submatrix multiplications; assume the output tile size is (*ε*, *η*, *ν*), using new coordinates $\left(i,\tilde{x},\tilde{y},\tilde{z}\right)$ to replace (*i*, *x*, *y*, *z*), yielding

(9) $$\begin{array}{c} M_{k,i,\tilde{x},\tilde{y},\tilde{z}}^{\varepsilon ,\eta ,\nu }=\overset{C-1}{\underset{c=0}{\sum }}U_{k,c}^{\left(\varepsilon ,\eta ,\nu \right)}V_{c,i,\tilde{x},\tilde{y},\tilde{z}}^{\left(\varepsilon ,\eta ,\nu \right)}. \end{array}$$

This equation represents the matrix multiplication, and it can be simplified as follows:

(10) $$\begin{array}{c} M^{\left(\varepsilon ,\eta ,\nu \right)}=U^{\left(\varepsilon ,\eta ,\nu \right)}V^{\left(\varepsilon ,\eta ,\nu \right)}. \end{array}$$

Algorithm 2 gives the overview of the 3D WMFA. The algorithm mainly consists of four stages, which are Winograd transformation of the input feature tile; Winograd transformation of the filter tile; the matrix multiplication, which is converted from the dot product of the transformed input tile and the transformed filter tile; and the inverse Winograd transformation of the result of the matrix multiplication.

### 3.3. Arithmetic Complexity Analysis

For input feature maps with size *N* × *C* × *D* × *H* × *W*, filters with size *K* × *C* × *k* × *k* × *k*, and the output features with size *N* × *K* × *M* × *P* × *Q*, the total number of float operations in the multiplication stage can be represented as follows:

(11) $$\begin{array}{c} L_{1}=2N\left\lceil \;\frac{M}{m}\;\right\rceil \left\lceil \;\frac{P}{m}\;\right\rceil \left\lceil \;\frac{Q}{m}\;\right\rceil CK{\left(\;m+r-1\;\right)}^{3}, \end{array}$$

where *r* is the filter size and *m* is the size of the output subtile. However, if we use the direct convolution method, which is computed according to the definition of convolution, the total number of float operations is computed as follows:

(12) $$\begin{array}{c} L_{2}=2NMPQCKr^{3}. \end{array}$$

Dividing *L*_1_ by *L*_2_ yields

(13) $$\begin{array}{c} \frac{L_{2}}{L_{1}}=\frac{m^{3}*r^{3}}{{\left(m+r-1\right)}^{3}}. \end{array}$$

Assuming *m* = 2 and *r* = 3, there is an arithmetic complexity reduction of (2*∗*2*∗*2*∗*3*∗*3*∗*3)/(4*∗*4*∗*4) = 216/64 = 3.375. However, there are some extra computations in Winograd transformation stage, so we cannot achieve so much complexity reduction in reality. The detailed performance improvements are shown in Section 5.2.

## 4. Implementation and Optimizations

### 4.1. Implementation

**Figure 1:**
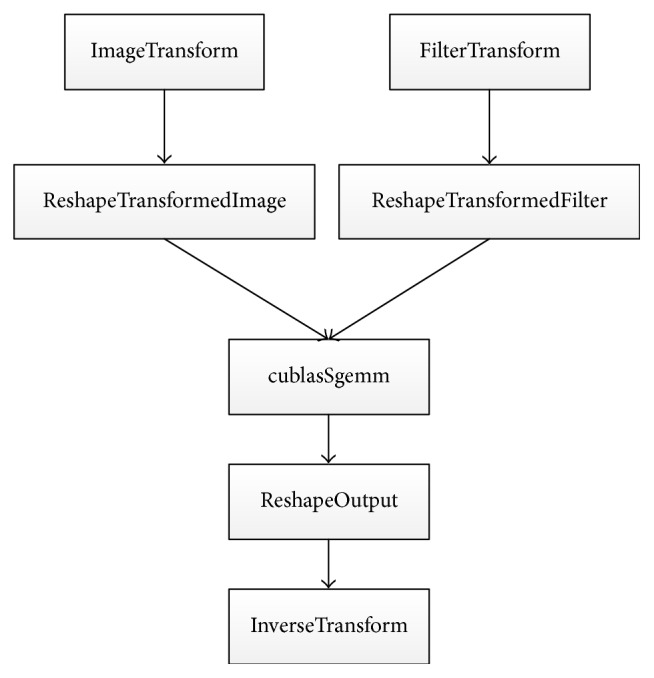
The computing flow of 3D WMFA.

We have three implementation versions for the 3D WMFA on a GPU. In our implementations, cuBLAS is called to do the multiplication. Furthermore, we manually implement six kernels according to Algorithm 2.  Figure 1 shows the flow of our baseline implementation. The* imageTransform* kernel transforms all the image subtiles, the* filterTransform* kernel transforms all the filter tiles, and* ouputTransform* kernel inversely transforms the result of the multiplication. For the baseline implementation, we also have two additional kernels to reorganize the transformed image data and transformed filter data and one kernel to reorganize the result of the multiplication before the results go to the* outputTransform* kernel.

Winograd transformation algorithm is suitable for parallelization on GPU. Taking the* imageTransform* kernel as an example, the input to the* imageTransform* kernel is the input feature map. We assume the input feature maps have a size of *N* × *C* × *D* × *H* × *W*, where *N* is the batch size, *C* is the number of input channels, and *D* × *H* × *W* is the size of a single channel. As is described in Algorithm 2, the number of image tiles for each channel is *P*, so the total number of image tiles is *P* × *C*; all those image tiles can be transformed independently. For the baseline of the GPU implementation, the image data is stored in* NCDHW* order, and we set the number of threads in one block to be 32, each thread is responsible for processing Winograd transformation of one input subtile, and the number of blocks in the grid is set to (⌈*N*/32⌉, ⌈*M*/*m*⌉⌈*P*/*m*⌉⌈*Q*/*m*⌉, *C*). We can make full use of the large-scale parallel processing units of a GPU when the number of blocks is large.

**Figure 2:**
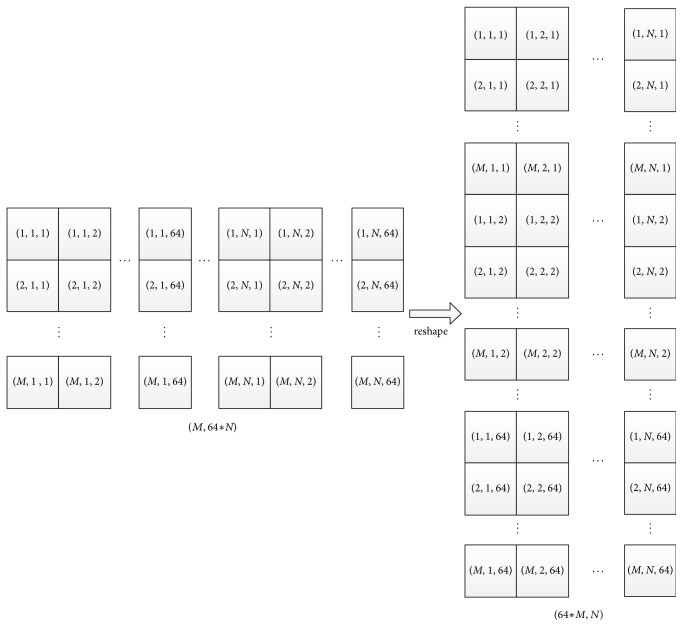
For an input matrix, its size is (*M*, *N∗α∗α∗α*); *α* is the tile size, here equal to 4. After the reshape kernel is applied, lots of small submatrices with new layouts are generated.

For the* filterTransform* kernel, there are *K* × *C* filter tiles; we still set the number of threads of one block to be 32 and the number of blocks to (⌈*K*/32⌉, *C*, 1). Before we call cuBLAS to implement the matrix multiplication, we need to reorganize the transformed filter and transformed image data. For transformed filter, there are *K* × *C* tiles in total, and each tile has size of *α* × *α* × *α*, each time we gather one value from one tile to generate a submatrix of size *K* × *C*.  Figure 2 shows how the transformed data are reorganized in new layout on GPU; they are implemented by the kernels* reshapeTransformedImage* and* reshapeTransformedFilter* in our baseline implementation. They gather correlated and transformed filters and transformed image data to form two submatrices, and* SGEMM* from the cuBLAS library is called to do the multiplication. The result of the multiplication also needs to be reshaped before the inverse transformation.

### 4.2. Optimizations

We make two optimizations to achieve higher performance. The first optimization is to align memory access, which can make memory access more efficient and increase the cache hit rate. The second optimization is to combine the transformation kernel with the reshape kernel to reduce global memory access.

The storing order of data and how data is accessed by a thread can significantly affect the performance. For the baseline implementation, the image data is stored in* NCDHW* order, and threads in the same block access data along the *N*-dimension, which means data accessed by threads keeps long distances. However, the size of the cache on a GPU is limited; therefore, if the distance between the data items accessed by threads is larger than the size of the cache line, then each thread needs to access global memory separately, causing lots of memory accesses. In our first optimization version, we change the storage order of image data to* CDHWN*. Since the image data is stored starting from the N-dimension, data accessed by all threads in the same block is continually stored, and all data items loaded from the global memory are useful, which means the bandwidth is fully used. The same optimization method is applied to the filter transformation and inverse transformation kernel.

Based on the first optimization, we apply our second optimization to improve performance further. The second optimization is to reduce the number of global memory accesses. For the baseline implementation, after the filter transformation or image transformation kernel is executed, the transformed filter or transformed image data need to be stored in a new layout using* reshapeTransformedFilter* and* reshapeTransformedImage* kernel and using the* ReshapeOutput* kernel before the inverse transformation, while on our second optimized version, we move the work of the reshape kernel to the transformation kernel, so in the optimized transform kernel, after the inputs are transformed, the result will be stored directly back in the expected layout. In the optimized inverse transformation, before inverse transformation, the required data is gathered directly from the global memory.

## 5. Experiments

### 5.1. Experimental Setup

All experiments are evaluated on a GeForce GTX 1080 GPU, which has a total amount of 8 GBytes global memory and has 20 multiprocessors. Detailed parameters of the GTX 1080 are shown in Table 1.

### 5.2. Performance Evaluation

We apply our 3D WMFA to a widely used 3D neural network called v3d [9], which is used to classify videos. The 3D neural network has five convolutional layers; Table 2 shows the information about these 3D convolutional layers.

**Figure 3:**
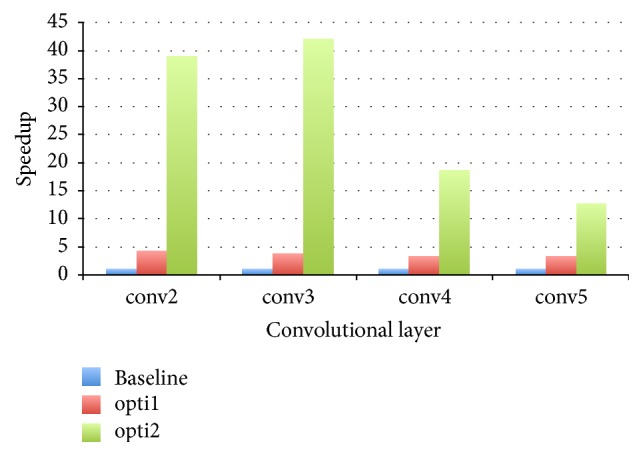
Speedup with different optimizations on 3D convolution layers.

Firstly, we evaluate the performance of our three implementations on the 3D convolutional layers, except for the first convolutional layer, which has only three input channels and is not yet supported in the algorithm.  Figure 3 shows the increase in speed we achieved after we used two optimizations. For the first optimization, we observe a 3 to 4 times speeding up for all these test convolution layers compared to the baseline implementation. However, for the second optimization, the maximum speeding up can be close to 42 for the third convolution; even the minimum speeding up is about 13 for the last convolution layer. The first optimization makes memory access more efficient, and the second optimization reduces lots of unnecessary global memory accesses. Since the latency of global memory access on a GPU is large, we achieve a good performance improvement in the second optimization.

**Figure 4:**
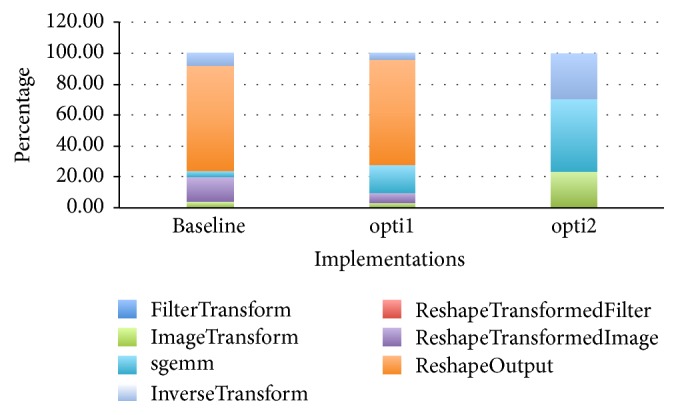
Time percentage distribution of each kernel in each implementation version for a specific convolution layer.

We explore the detailed performance for one specific convolution layer to see how these two optimizations improve each kernel separately. We profile the time percentage of each kernel in each implementation version for conv3, which takes most of the computation time among all these convolution layers.  Figure 4 shows the profiling results; the kernel* ReshapeOutput* in both baseline and the first optimization takes up the most time, since the kernel contains lots of global memory accesses. However, in the second optimization version, there are no* reshape* kernels and the kernel* sgemm* takes most of the execution time. In all three implementations, the kernel* filterTransform* takes only about 0.1% of the total time.

**Figure 5:**
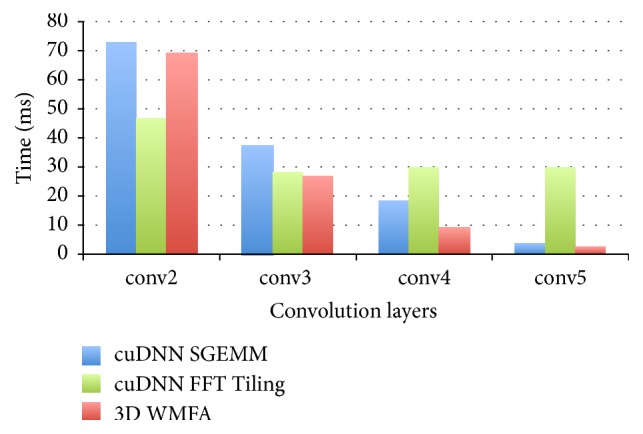
Execution time of different methods on 3D convolution layers.

Finally, we compare our best optimized implementation with the cuDNN library. The cuDNN library is the fastest deep-learning library. There are two algorithms available to implement a 3D convolution layer: one converts the convolution to a matrix multiplication and the other exploits the FFT tiling method to implement the convolution. We use* cuDNN SGEMM* and* cuDNN FFT Tiling* to represent the two methods called in the cuDNN library, and we use 3*D WMFA* to represent our algorithm.  Figure 5 shows the execution time of these three methods on four convolution layers. The 3*D WMFA* method is about 30% slower than the* cuDNN FFT Tiling* method on the conv2 layer; however, it is a bit faster than* cuDNN SGEMM* method. The* cuDNN FFT Tiling* method achieves a fast speed at the cost of consuming a large amount of memory. Since parameters *C* and *K* are not large in the conv2 layer, this makes the matrix multiplication in 3*D WMFA* on small scale, which affects the performance. However, with parameters *C* and *K* increased on layers conv3, conv4, and conv5, the 3*D WMFA* method achieves better performance than the other two methods. We added the execution time of each layer for each method, the total time of all layers is 132.6 ms for cuDNN SGEMM method, 135.4 ms for cuDNN FFT Tiling method, and 108.2 ms for 3D WMFA which is better than the other two methods.

We can also calculate the performance of these two methods in* TFLOPS*.  Table 3 shows the effective* TFLOPS* of* cuDNN SGEMM* and 3*D WMFA* method. We achieve a maximum speedup of 1.96 compared to* cuDNN SGEMM*.

## 6. Conclusions

A 3D convolution layer requires a high computational cost and consumes lots of memory. We designed a 3D WMFA to implement 3D convolution operation. Compared to traditional convolution methods, such as SGEMM or FFT, the 3D WMFA can reduce computation, in theory. When we implemented the algorithm on a GPU, we observed the expected performance of the algorithm in the experiments. For some 3D convolution layers, we even achieve close to 2 times speedup compared to cuDNN library.

However, the computation and memory requirements of 3D convolution obviously increase with more complex 3D neural networks. In our future work, we will implement *F*(4 × 4 × 4,3 × 3 × 3) to reduce the computation further to ease the intensive computation problem and adopt a FP16 data type to compute, which can save half of the memory usage directly. It is also necessary to parallel the convolution computation among multi-GPUs or multinodes.

## Figures and Tables

**Algorithm 1:**
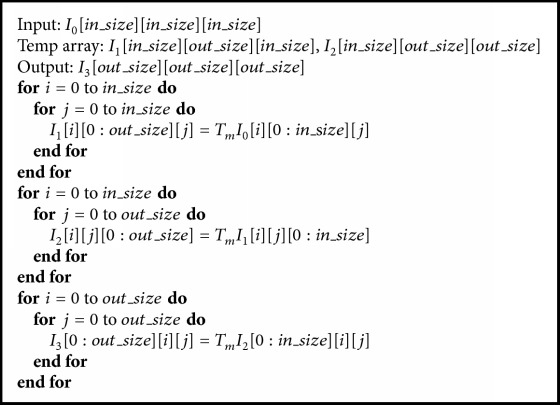
3D winograd transformation.

**Algorithm 2:**
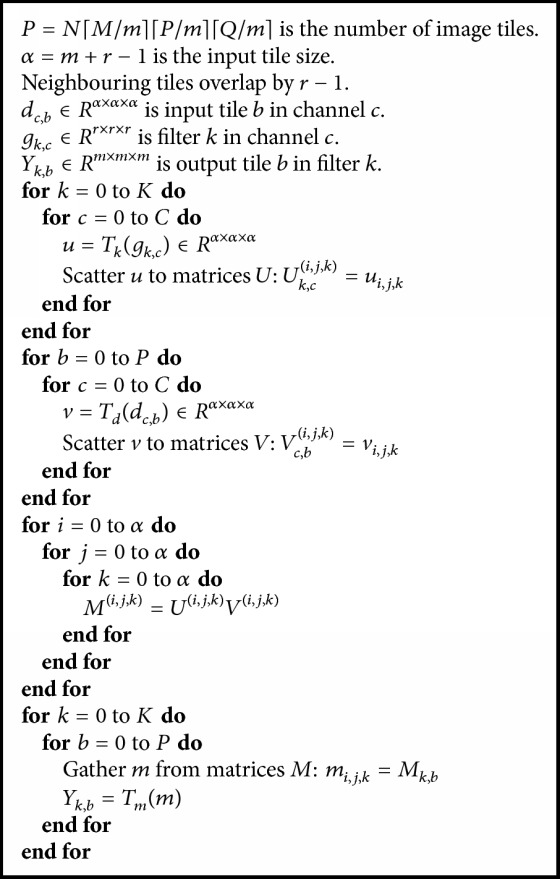
3D Convolutional layer implemented with WMFA *F*(*m* × *m* × *m*, *r* × *r* × *r*).

**Table 1:** Properties of the GeForce GTX 1080.

Parameters	Values
CUDA capability major/minor version number	6.1
Total amount of global memory	8 GB
CUDA cores	2560
L2 cache Size	2 MB
Warp size	32
Total number of registers available per block	64 KB

**Table 2:** Convolution layers of a 3D network; the filter size in all layers is 3 × 3 × 3, and the GFLOPS columns calculate the number of flops operations in each convolutional layer. Assume the batch size is 32.

Layer	*C* × *D* × *H* × *W* × *N*	*K*	GFLOPS
conv1	3 × 16 × 112 × 112 × 32	32	16.65
conv2	32 × 16 × 56 × 56 × 32	64	88.8
conv3	64 × 8 × 28 × 28 × 32	256	88.8
conv4	256 × 4 × 14 × 14 × 32	256	44.4
conv5	256 × 2 × 7 × 7 × 32	256	5.55

**Table 3:** Performance of cuDNN SGEMM versus that of the 3D WMFA on 3D convolution layers. Performance is measured in effective TFLOPS.

Layer	*C* × *D* × *H* × *W* × *N*	*K*	TFLOPS		Speedup
			cuDNN SGEMM	3D WMFA	
conv2	32 × 16 × 56 × 56 × 32	64	1.21	1.28	1.05
conv3	64 × 8 × 28 × 28 × 32	256	2.38	3.31	1.39
conv4	256 × 4 × 14 × 14 × 32	256	2.4	4.72	1.96
conv5	256 × 2 × 7 × 7 × 32	256	1.46	2.1	1.44
